# DNA damage (8-OHdG) and telomere length in captive Psittacidae birds with different longevity

**DOI:** 10.3389/fvets.2024.1430861

**Published:** 2024-08-07

**Authors:** Angélica Domínguez-de-Barros, Inés Sifaoui, Roberto Dorta-Guerra, Jacob Lorenzo-Morales, Rafael Castro-Fuentes, Elizabeth Córdoba-Lanús

**Affiliations:** ^1^Instituto Universitario de Enfermedades Tropicales y Salud Pública de Canarias (IUETSPC), Universidad de La Laguna, San Cristóbal de La Laguna, Spain; ^2^Centro de Investigación Biomédica en Red de Enfermedades Infecciosas (CIBERINFEC), Instituto de Salud Carlos III, Madrid, Spain; ^3^Departamento de Matemáticas, Estadística e Investigación Operativa, Facultad de Ciencias, Sección de Matemáticas, Universidad de La Laguna, San Cristóbal de La Laguna, Spain; ^4^Departamento de Obstetricia y Ginecología, Pediatría, Medicina Preventiva y Salud Pública, Toxicología, Medicina Legal y Forense y Parasitología, Facultad de Ciencias de la Salud, Sección Medicina, Universidad de La Laguna, San Cristóbal de La Laguna, Spain; ^5^Departamento de Ciencias Médicas Básicas, Facultad de Ciencias de la Salud-Sección Medicina, Universidad de La Laguna, San Cristóbal de La Laguna, Spain

**Keywords:** oxidative stress, 8-OHdG, DNA damage, telomeres, longevity, birds, Psittacidae

## Abstract

Aging is a complex process influenced by internal and external factors. Oxidative stress damages DNA, leading to 8-hydroxy-2’ deoxyguanosine formation (8-OHdG). Telomere shortening is considered a biomarker of aging and oxidative stress may enhance its attrition. The ability to manage and repair oxidative stress varies among species and life histories. Avian species, such as Psittacidae birds, exhibit exceptional lifespans despite their physiological characteristics that might suggest otherwise. This study investigates 8-OHdG levels in serum samples from long- and short-lived birds of the order Psittaciformes, examining their relationship with telomere length and antioxidant capacity based on lifespan strategies. Among 43 individuals analyzed 26 belonged to the “long-lived species” group and 17 belonged to the “short-lived species” one. Relative telomere length (rTL) was measured in DNA isolated from whole blood by qPCR, and oxidative stress markers, such as Total Antioxidant Capacity (TAC) and 8-OHdG, were determined by spectrophotometry in serum samples. Long-lived birds had longer rTL than short-lived ones [1.308 ± 0.11 vs. 0.565 ± 0.13, (*p* < 0.001)]. On the contrary, short-lived birds showed more DNA damage than their counterparts [3.847 ± 0.351 vs. 2.012 ± 0.308, respectively, (*p* < 0.001)]. Old birds had shorter rTL than young ones, for both longevity groups (*p* < 0.001). Although no correlation was found between 8-OHdG levels and age, nor 8-OHdG and telomere length, long-lived birds exhibited 75.42-unit increased TAC levels when increased 8-OHdG concentrations (*p* = 0.046). These findings highlight distinct patterns of telomere length and oxidative stress influenced by lifespan strategies among avian longevity groups.

## 1 Introduction

Aging is vital and complex and results over time in a loss of functional capacity and stress resistance of an organism, associated with an increased risk of morbidity and mortality. It is influenced by genetic factors, environment, and lifestyle, and affects, interconnectedly, all body tissues and organs (1). Oxidative stress causes damage to the primary biomolecules, including lipids, proteins, and DNA. Particularly, DNA damage is considered one of the most important in gerontology, as molecular repair depends on the information coded in the DNA (2). Continuous damage can accumulate over an individual’s lifespan, reducing cellular functioning and ultimately activating apoptosis mechanisms (3). The study of oxidative stress is a key piece in understanding diseases and physiological aging (4, 5).

One of the consequences of DNA damage is the formation of 8-hydroxy-2′ deoxyguanosine (8-OHdG). It is formed when reactive oxygen species (ROS) act on DNA strands, adding radical DNA bases and generating a variety of new oxidation products (6, 7). Several authors reported the increase of this molecule with normal aging in organs, and in different stages of diseases, such as Parkinson’s disease, diabetes, cystic fibrosis, and muscular dystrophy (8). 8-OHdG has been proposed as an adequate and ubiquitous biomarker of oxidative stress. During the reparation of damaged DNA, 8-OHdG is excised and excreted further in metabolisms so that it can be measured in plasma and urine (3).

Another main marker of aging is telomere shortening. Telomeres are protective DNA regions at the end of chromosomes, containing 5–15 kb of (TTAGGG)n repeats, that prevent degradation and recombination and support chromosomal stability. Oxidative stress can cause telomere shortening besides cell division-dependent natural processes (1, 9). Telomeres are more predisposed to suffer DNA damage due to their enriched G-regions and their reduced repair capacity, so ROS-induced damage can rapidly accumulate in telomeres (10). Indeed, DNA damage accumulation with aging and telomere shortening may be related processes (11). When telomeres reach a critical length, cell death, and apoptotic mechanisms are activated.

The ability of organisms to cope with oxidative stress depends first on the damage level, and then on their capacity to invest in its repair, which varies between different species, life histories, and age-related changes (12). Interestingly, some avian clades are considered long-lived and possibly resistant to aging processes despite their physiological characteristics, which would indicate otherwise (13–15). Psittacidae birds in particular are considered among the most long-living birds, with some exceptional maximum longevity records (16).

Previous studies from our group have found that long-lived psittacine show greater initial telomere length which is maintained over time compared to their short-lived counterparts, and also display a better whole antioxidant status. On the contrary, short-lived birds show higher levels of oxidative damage accumulated in lipids (17). Still, little information exists regarding the measurement of 8-OHdG and its relationship with telomere length dynamics in Psittacidae birds.

In the present study, we aim to investigate the variation of 8-OHdG levels in serum samples of psittacine birds with long and short lifespans. We intend to analyze the relationship between this DNA damage marker, telomere length, and antioxidant capacity depending on the life strategies of these animals.

## 2 Methods

### 2.1 Sample selection

From a total of 81 birds of the order Psittaciformes analyzed in the Psittacine longevity project (2019) (17), a subsample of 43 individuals was evaluated in the present study due to sample availability. Of these, 26 belonged to the “long-lived species” group (species *Amazona barbadensis*, *Anodorhynchus hyacinthinus*, *Cacatua moluccencis*, and *Ara macao*), and 17 belonged to the “short-lived species”one (species *Agapornis taranta, Psitteuteles goldiei*, and *Trichoglossus johnstoniae*). Individuals were categorized as “young” and “old,” both for long- and short-lived birds, considering maximum longevity and the time they end the pre-puberty/ puberty phase and reach maturity according to the reported literature research (18) (Supplementary Table 1).

Psittacine birds included in this study are preserved in captivity in optimal conditions in Loro Parque Fundación research facilities, in Tenerife, Spain. This guarantees no crossed influence of external factors such as predation, diet, or extreme environmental factors.

Blood extraction was performed by venipuncture from the right jugular vein, acting properly as indicated by ARRIVE guidelines. The procedure for sample process and storage was already reported by our group (17).

### 2.2 Biomarkers assay

DNA was isolated from 5 μl of blood using the Dneasy Blood & Tissue Handbook kit (Qiagen) following manufacturers’ instructions. Telomere length was assessed using the real-time PCR (qPCR) procedure as detailed previously (17) based on the studies by Cawthon (19), and Criscuolo et al. (20). Relative telomere length (rTL) is expressed as the T/S ratio, measuring the relative amount of telomeric repeats of the *Tel* gene (T), versus (vs.) the *GAPDH* single-copy control gene (S) using the 2^(−∆CT)^ formula. The qPCR assay was set up in a StepOne Plus thermocycler (AppliedBiosystems, ThermoFisher Scientific, MA, USA). All samples were run in duplicates.

The measurement of antioxidant capacity (TAC) in serum samples was performed as detailed previously (17). Each sample was evaluated in duplicate, and absorbance was quantified at 593 nm on the EnSpire Multimode Plate Reader (Perkin Elmer, Madrid, Spain).

The determination of the DNA damage marker, 8-hydroxy-2′ -deoxyguanosine (8-OHdG), was assessed using the competitive enzyme immunoassay OxiSelectTM Oxidative DNA Damage ELISA Kit (8-OHdG Quantitation; CellBiolabs, San Diego, California) following the manufacturer’s instructions. Serum samples were 1:20 diluted in Assay Diluent and then used 50 μl in the assay. The absorbance was determined spectrophotometrically at 450 nm on the EnSpire Multimode Plate Reader (Perkin Elmer, Madrid, Spain). The 8-OHdG quantitation (ng/μl) was determined by the comparison from a 4-parameter-logistic line Standard Curve, using SigmaPlot 12.0 program (Systat Software, 2010 Inc., San Jose, California).

### 2.3 Statistical analysis

Continuous variables were described using means and standard deviation (SD) or standard error of the mean (SEM) as appropriate. Due to the age range distribution of this subset of individuals, statistical tests regarding age have been used with the categorization of this variable in “young/old” for both longevity groups (in long-lived birds young<15 years; old>15 years; and for short-lived birds young<5 years; old>5 years).

To assess the impact of relative telomere length (rTL) and DNA damage on age and longevity, Linear Mixed Effects Models were applied, considering longevity and the “young/old” classification as fixed factors, and species as random factor. Regarding the evaluation of 8-OHdG in long-lived individuals, the species *Anodorhynchus hyacinthinus* was excluded from the analysis due to only having old individuals. To explore the association of Total Antioxidant Capacity (TAC) with DNA damage and age across different longevities, Linear Mixed Effects Models were applied, incorporating DNA damage as a covariate, the “young/old” variable as a fixed factor, and species as a random factor.

All statistical analyses were performed by using SPSS v.25.0 (IBM Statistics) and two-tailed *p*-values<0.05 were considered significant. Graphs were designed with GraphPad Prism v9.0 (Dotmatics, GraphPad Software, San Diego, California United States).

## 3 Results

**Figure 1:**
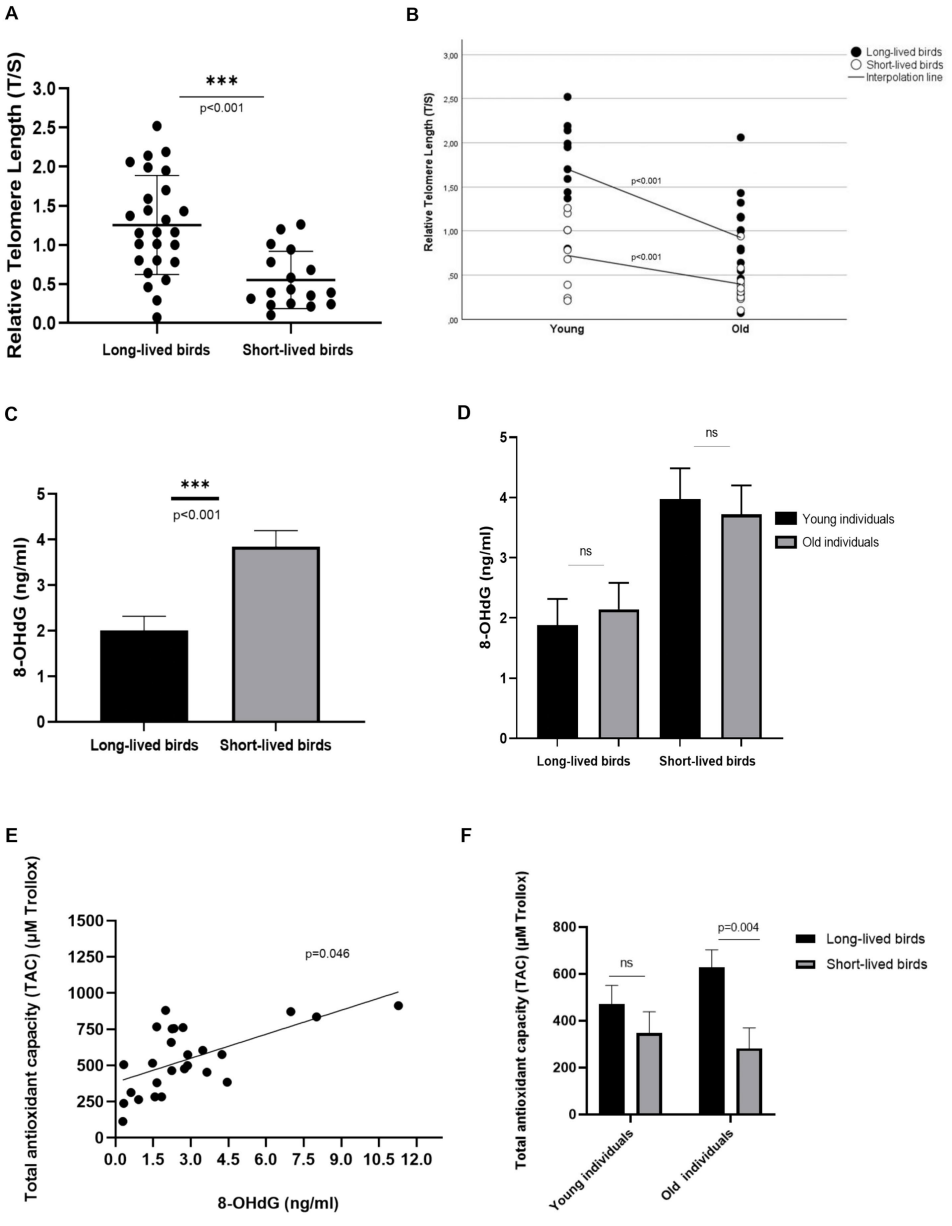
Relative Telomere Length (rTL) in the subset of individuals analyzed in the study. **(A)** rTL in long- and short-lived birds. **(B)** Correlation between rTL and age in long- and short-lived birds **(C)** 8-OHdG concentration in long- and short-lived psittacine birds **(D)** 8-OHdG concentration in young and old individuals of the different longevity groups included in this study; ns: non-significant. **(E)** Correlation between the levels of the DNA damage marker, 8-OHdG, and the Total Antioxidant Capacity (TAC) present in long-lived birds. **(F)** Total Antioxidant Capacity (TAC) in long- and short-lived birds of different ages.

In this sample subset, long-lived birds had longer rTL than short-lived ones [1.308 ± 0.11 vs. 0.565 ± 0.13, (*p* < 0.001), respectively]. Also, a correlation with age was found in the analyzed individuals, as young individuals had longer rTL than those considered old, both for long- [1.693 ± 0.16 young vs. 0.923 ± 0.14 old, (*p* < 0.001)] and short-longevity birds [0.732 ± 0.18 young vs. 0.398 ± 0.17 old, (*p* < 0.001)]; (Figures 1A,B).

Short-lived birds accumulated more DNA damage, by showing 1.835 (IC95% 0.886–2.783) increased levels of 8-OHdG than long-lived birds [3.847 ± 0.351 vs. 2.012 ± 0.308, respectively, (*p* < 0.001)]; (Figure 1C). When analyzing this oxidative marker concerning the different age groups, similar levels of 8-OHdG were found in the studied age groups, both for long-lived [1.879 + 0.44 young vs. 2.145 + 0.44 old] and for short-lived birds [3.974 + 0.5 young vs. 3.720 + 0.48 old; (*p* = 0.989)]; (Figure 1D).

No relation was found between increased levels of 8-OHdG in serum and relative telomere length observed in these birds, nor for those within the long-lived and short-lived group, (*p* = 0.969 respectively); (Supplementary Figures 1, 2).

On the other hand, increased 8-OHdG concentrations correlated with the high Total Antioxidant Capacity (TAC) levels observed in long-lived birds. We found a 75.42-unit increase in TAC levels (95% CI 1.571–149.373) when increased DNA damage (*p* = 0.046); (Figure 1E). This relationship was not observed in short-lived birds (*p* = 0.335). Long-lived birds also showed higher TAC than short-lived birds in these analyzed individuals (*p* = 0.037). Differences in TAC levels were found when analyzing old individuals; old long-lived birds showed 346.86 units of Trollox (μM) [IC 95%: 115.14 -578.58] more than old short-lived birds (*p* = 0.004); (Figure 1F).

## 4 Discussion

Several studies have focused on studying the underlying mechanisms and potential biomarkers of aging in various species with different lifespans, including mammals, birds, and other groups. However, little attention has been paid to exploring oxidative stress, especially that directly targeting DNA damage in Psittacidae species with different lifespans. In this research, a novel and comprehensive study of the variation in DNA damage according to the longevity strategy and age of these birds has been conducted. Moreover, this study has examined the impact of this damage concerning telomere length in these birds and their defense mechanisms. In this study, besides confirming what was previously reported by our group, on short-lived birds having shorter rTL (17), we found that this group also exhibited increased levels of DNA damage than long-lived ones.

Telomeric DNA sequences are plausibly more susceptible to oxidative stress due to their high guanine residue content, especially by the formation of 8-OHdG (21). At a cellular level, it is well-accepted that oxidation compromises telomere length and causes telomere attrition through a variety of mechanisms. Research has confirmed that DNA repair capacity declines with age, causing the accumulation of unresolved or miss-repaired DNA damage, resulting in increased telomere shortening and an overall pathology (22, 23). Several studies have evaluated the relationship between oxidative damage and age in birds but obtained different results (12, 14). Following this, we found a tendency of increased 8-OHdG levels in old individuals in contrast to younger ones, within long-lived birds.

It is possible that in long-lived species, the increase of oxidative stress is not linearly accumulated, being only evident in specific stages of their life (24). Also, the measurement of 8-OHdG in serum can reflect concentrations due to both damage and repair processes (12), masking each other’s effect.

Oxidative damage could be repaired *in vivo* through different processes (21). In species with shorter lifespans, this damage could remain consistently high throughout their lives so we could not assess a clear relationship with age, the same was observed for other oxidative markers of oxidative stress, such as lipid peroxidation products, which also accumulate in short-lived birds (17), suggesting a systemic level of oxidative damage.

Recent research has shown complex interactions between reactive molecules, oxidative stress, and telomere dynamics (25). As might be expected, birds or other individuals with high longevity, having longer telomeres, would be predisposed to suffer greater damage (26). However, a relationship between telomere length and DNA damage of long-lived birds could not be found for any of the longevity groups of birds included in this study, although higher levels of nuclear 8-oxo-deoxyguanosine have been shown to correlate with shorter telomeres (11). Further studies are needed to assess how different types of DNA damage correlate, and how this DNA damage correlates with telomere shortening rates (11).

This can be also explained by the different strategies that these animals own to solve the costs of oxidative stress and produce molecules that are traded off for other self-maintenance processes (10). Some ROS molecules are neutralized by innate antioxidants, such as superoxide dismutase (SOD), while other oxidant molecules may be scavenged by exogenous sources, dietary antioxidants, etc. (23). In previous studies of our group, a higher total antioxidant capacity was observed for long-lived parrots (17). In this study, we found that long-lived birds may response to high concentrations of 8-OHdG with elevated TAC concentrations. We hypothesize that these birds may use this general mechanism, along with other more specific ones, to counteract the damage suffered to their DNA. In agreement with the literature, elevated levels of plasma antioxidants have been also reported in similar studies (27). These results support a compensatory mechanism for the “challenges of aging” (28).

Taken all together, the processes of intercommunication and influence of oxidative damage to telomeres, and the reflection of these in the longevity of birds are complex, but there is a shred of intriguing evidence for differential and potentially advantageous mechanisms, especially in long-lived birds, that allow them to resist the detrimental effects of oxidative damage, that their short-lived counterparts fail in amend. In conclusion, long-lived birds show a greater telomere length and overall, less DNA damage than short-lived birds. Long-lived psittacine also showed higher total antioxidant capacity when facing increased levels of oxidative damage.

### 4.1 Strengths and impact of the study

In general, parrots typically show no signs of aging until they reach a certain advanced age, unless affected by an infectious disease. The principal explanation for this is that they have special physiological traits that help them survive, but also, evidence suggests that they might have evolved special molecular mechanisms, especially long-lived birds, to be protected against rapid aging (13).

Despite these notable characteristics, and that birds have been considered potential models for the study of aging for at least a decade for their possibly better resemblance to other long-lived studied animal models (29), more in-depth studies are not being carried out on parrots.

By studying these birds, which are raised under healthy and controlled conditions at the Loro Parque facilities, allows us to reduce the influence of external factors. In this way, we can provide a more accurate understanding of the effects of senesce in the studied biomarkers and how organisms respond depending on their longevity. To our knowledge, this study is the first to examine the impact of DNA damage on telomere length in Psittacidae birds with varying lifespans.

Psittacidae birds, particularly the long-lived ones, show promising results in having conserved strategies and mechanisms that support longevity, similar to what has been reported in other birds and animals (30, 31). Also, other mechanisms found helpful in other species need to be studied further in the Psittacidae family, such as the expression of certain genes involved in longevity, and mechanisms focused more on telomere maintenance over time rather than its elongation when damaged by ROS (32, 33).

### 4.2 Limitations of the study

The assessment of the oxidative stress biomarkers has been measured in serum samples, which reflect the systemic state of these markers in the individuals. The extent of the damage and time courses of the removal and repair may be very different, and the obtained results related to DNA damage in this study may not coincide with what is found in specific tissues. Even though this kind of measurement offers a non-invasive method and a very useful approach, the results need to be contrasted in further studies.

Specifically, the lack of significant correlations between the DNA damage markers, and telomere length and age are possible due to low sample individuals. Also, the cross-sectional nature of this study may not reflect the true relationship of these markers in the long run. There is a need to perform longitudinal studies covering a greater range of ages to complete the knowledge regarding the behavior of the 8-OHdG marker over time. Also, the assessment of multiple biomarkers to fully understand the dynamic interplay between ROS, ROS-damaged molecules, antioxidants, and repair mechanisms needs to be performed.

## 5 Conclusion

In conclusion, this study evaluated the relationship between oxidative damage, telomere dynamics, and longevity in different Psittacidae species. The findings revealed that long-lived birds exhibited greater telomere length and overall lower DNA damage levels (8-OHdG) than short-lived birds. Although not significant, a tendency for 8-OHdG to accumulate with age for long-lived birds is observed. Moreover, this study underscored the importance of antioxidant capacity particularly in long-lived parrots, where elevated concentration of TAC may act to mitigate the detrimental effects of oxidative stress, suggesting adaptive mechanisms that contribute to their extended lifespan. Nevertheless, further longitudinal research and the exploration of additional biomarkers are needed to fully elucidate the complex interplay between oxidative stress and telomere length in the Psittacidae aging process.

## Data availability statement

The original contributions presented in the study are included in the article/Supplementary material, further inquiries can be directed to the corresponding author.

## Ethics statement

Ethical approval was not required for the study involving animals in accordance with the local legislation and institutional requirements because biological samples were taken as part of the routine annual veterinary control without any harm or risk to the life of the animals under study at Loro-Parque Fundación. Animals were treated appropriately according to the Spanish legislation and the EU Directive (2010/63/UE) on “Protection of Animals Used for Experimental and Other Scientific Purposes”. The study complies with the ARRIVE guidelines developed by the NC3Rs and all efforts were made to minimize the number of animals used to produce reliable scientific data, as well as animal suffering.

## Author contributions

AD-d-B: Data curation, Formal analysis, Investigation, Methodology, Writing – original draft, Writing – review & editing. IS: Formal analysis, Methodology, Writing – original draft. RD-G: Data curation, Formal analysis, Writing – review & editing. JL-M: Funding acquisition, Writing – review & editing. RC-F: Conceptualization, Funding acquisition, Investigation, Writing – original draft, Writing – review & editing. EC-L: Conceptualization, Formal analysis, Funding acquisition, Investigation, Methodology, Project administration, Supervision, Validation, Writing – original draft, Writing – review & editing.
