# Supplementary material for: DNA damage (8-OHdG) and telomere length in captive Psittacidae birds with different longevity
Source: Front Vet Sci. 2024 Aug 7;11:1430861. doi: 10.3389/fvets.2024.1430861 (PMC11335655; doi:10.3389/fvets.2024.1430861)
Supplement: Supplementary file 1 [file Data_Sheet_1.docx]

Supplementary Material

DNA Damage (8-OHdG) and telomere length in captive Psittacidae birds with different longevity

**Domínguez-de-Barros, Angélica ^1^; Sifaoui, Inés ^1,2^; Dorta-Guerra, Roberto ^1,3^; Lorenzo-Morales, Jacob ^1,2,4^; Castro-Fuentes, Rafael ^┼5^; Córdoba-Lanús, Elizabeth ^┼1,2*^**

*** Correspondence:** Elizabeth Córdoba-Lanús

[acordoba@ull.edu.es](mailto:acordoba@ull.edu.es)

# Supplementary Data

# Supplementary Table 1. Characteristics of long-lived and short-lived species of the selected psittacine birds in this study.

| **Longevity Group** | **Species** | **Young Individuals ^a^ (<15 years)** | **Maturity status^b^** | **Old Individuals (>20 years)** | **Maturity status** |
| --- | --- | --- | --- | --- | --- |
|  | *Amazona barbadensis* | 29957 (3 yrs) | immature | 232 (29 yrs) | mature |
|  |  | 30038 (3 yrs) | immature | 377 (28 yrs) | mature |
|  |  | 29990 (3 yrs) | immature | 8330 (21 yrs) | mature |
|  |  | 30039 (3 yrs) | immature |  |  |
|  |  |  |  |  |  |
|  | *Anodorhynchus hyacinthinus* | - |  | 4690 (24 yrs) | mature |
|  |  | - |  | 5820 (23 yrs) | mature |
| **Long-lived group** |  | - |  | 29906 (28 yrs) | mature |
|  |  | - |  | 3708 (25 yrs) | mature |
|  |  |  |  |  |  |
|  | *Cacatua moluccensis* | 21699 (10 yrs) | young mature | 787 (35 yrs) | mature |
|  |  | 22860 (9 yrs) | Immature | 2072 (28 yrs) | mature |
|  |  | 19001 (12 yrs) | young mature | 3281 (25 yrs) | mature |
|  |  |  |  | 5606 (23 yrs) | mature |
|  |  |  |  | 1777 (35yrs) | mature |
|  |  |  |  |  |  |
|  | *Ara macao* | 27237 (6yrs) | immature | 26 (36yrs) | mature |
|  |  | 27387 (6yrs) | immature | 2316 (29yrs) | mature |
|  |  | 16763 (14 yrs) | young mature | 921088 (27 yrs) | mature |
|  |  | 26636 (6 yrs) | immature |  |  |
| Total |  | 11 |  | 15 |  |
| **Longevity Group** | **Species** | **Young Individuals  (<5 years)** | **Maturity status** | **Old Individuals (>5 years)** | **Maturity status** |
|  | *Agapornis taranta* | 31414 (1 yrs) | immature | 27500 (7 yrs) | mature |
|  |  | 31415 (1 yrs) | immature | 27501 (7 yrs) | mature |
|  |  |  |  | 27497 (7 yrs) | mature |
|  |  |  |  |  |  |
|  | *Psitteuteles goldiei* | 31445 (1 yrs) | immature | 25114 (7 yrs) | mature |
|  |  | 31454 (1 yrs) | immature | 25499 (7 yrs) | mature |
| **Short-lived group** |  | 31347 (1 yrs) | immature | 29347 (7 yrs) | mature |
|  |  | 31545 (1 yrs) | immature |  |  |
|  |  | 31741 (1 yrs) | immature |  |  |
|  |  |  |  |  |  |
|  | *Trichoglossus johnstoniae* | 30703 (2 yrs) | immature | 28818 (9 yrs) | mature |
|  |  |  |  | 26336 (7 yrs) | mature |
|  |  |  |  | 25257 (7 yrs) | mature |
| Total |  | 8 |  | 9 |  |

a) Identification code of the individuals selected for this study and their age at the time of sampling. Provided by Loro Parque Fundación.

b) Breed information from AnAge database (<https://genomics.senescence.info/species/index.html>) and ZIMS Species 360 Global Information Serving Conservation database (<https://zims.species360.org/Login.aspx?ReturnUrl=%2f>). Categorized as “immature, young mature and mature” by the criteria chosen for the previous study of our group (Domínguez-de-Barros et al., 2023)

# Supplementary Figures


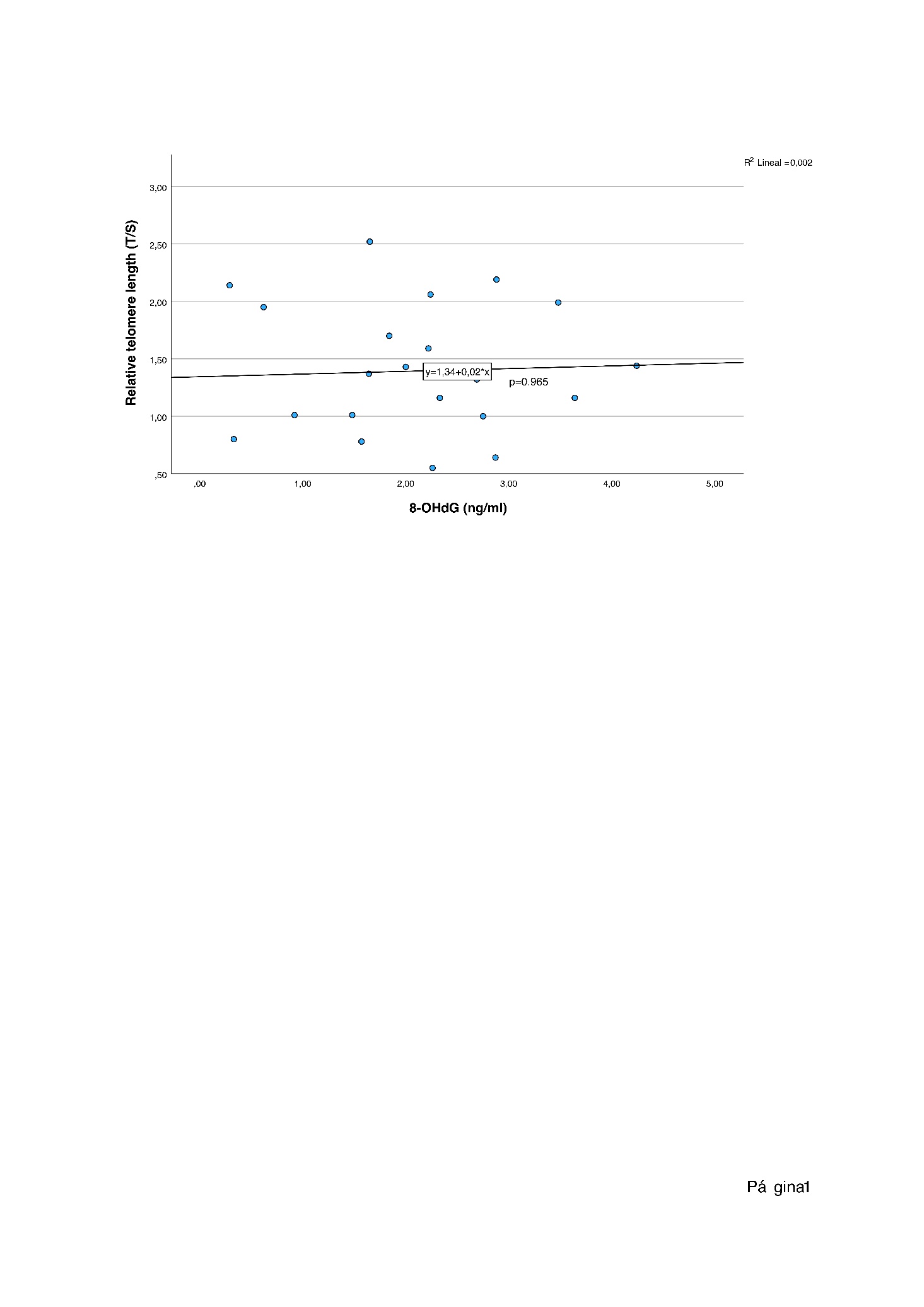


**Supplementary Figure 1.** Correlation between relative telomere length (rTL) and quantification of DNA damage products (8-OHdG) in long-lived birds.


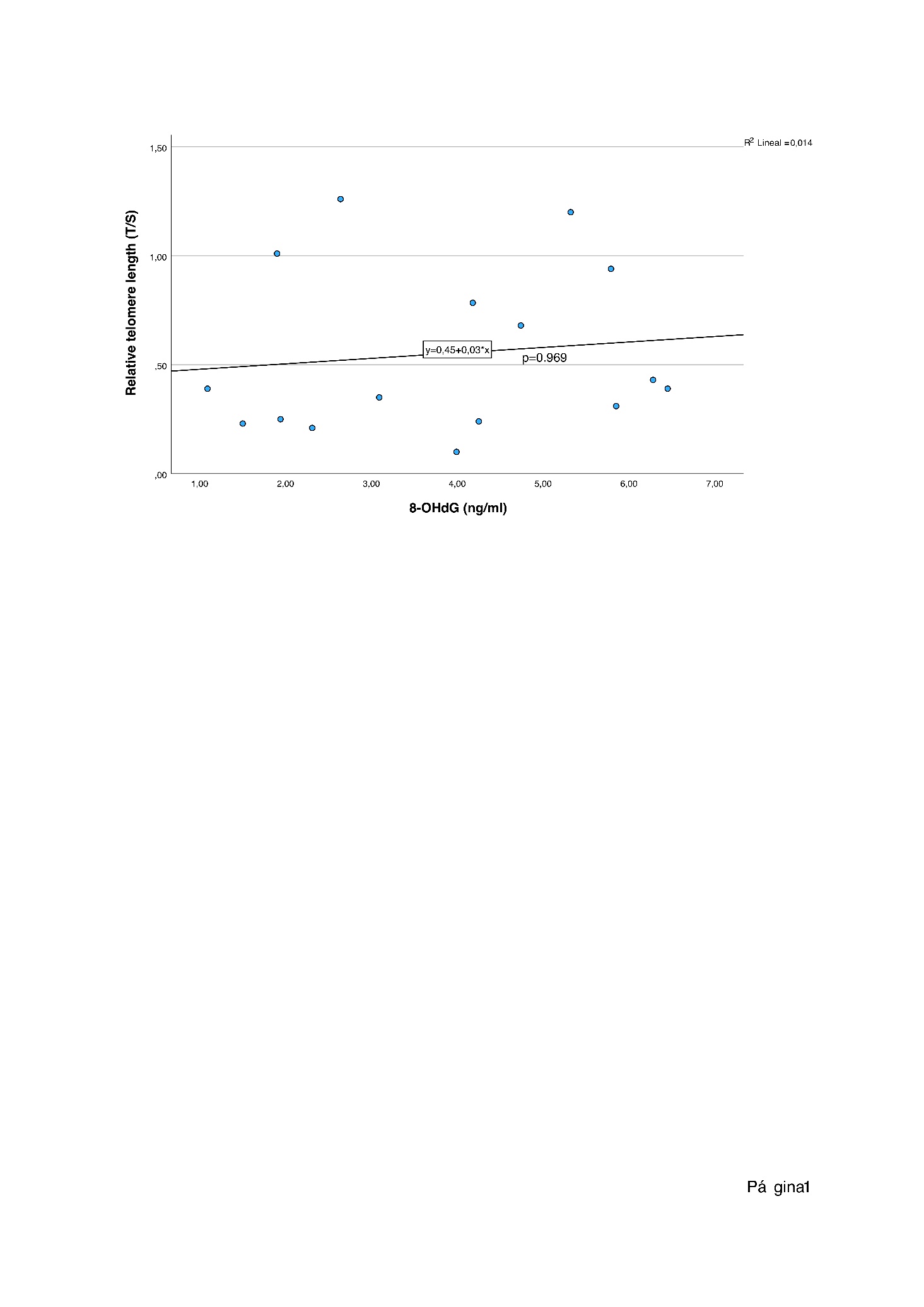


**Supplementary Figure 2.** Correlation between relative telomere length (rTL) and quantification of DNA damage products (8-OHdG) in short-lived birds.

**
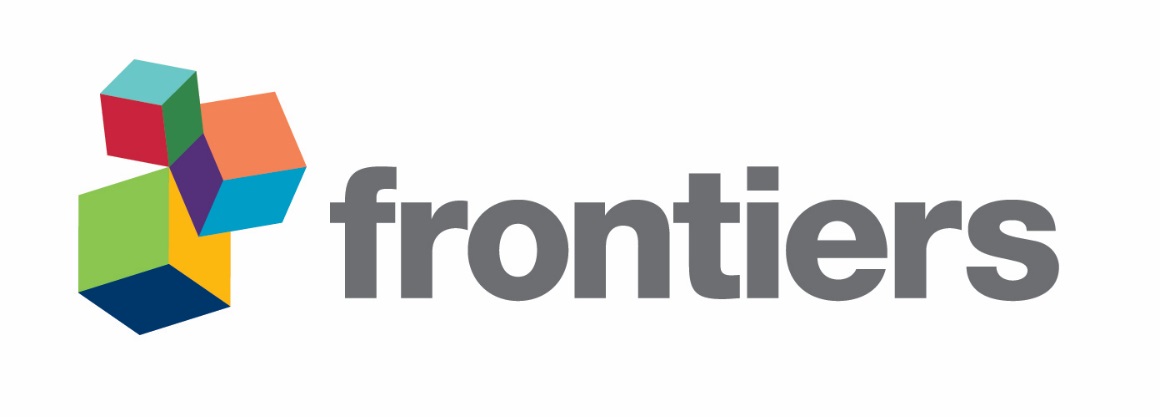
**
